# New Elastic Fiber Formation Can Be Induced by Intradermal Injection of Transparent Porcine‐Derived Type I Atelocollagen in Mice

**DOI:** 10.1111/jocd.71166

**Published:** 2026-08-31

**Authors:** Seung Min Oh, Min Seong Kim, Geebum Kim, Myungjune Oh

**Affiliations:** ^1^ ON Clinic Dermatology & Plastic Surgery Seoul Republic of Korea; ^2^ Department of Anatomy & Cell Biology, College of Medicine Gachon University Incheon Republic of Korea; ^3^ Functional Cellular Networks Laboratory, Lee Gil Ya Cancer and Diabetes Institute Gachon University Incheon Republic of Korea; ^4^ INEE Clinic Seoul Republic of Korea; ^5^ Misogain Dermatology Clinic Gimpo Republic of Korea

**Keywords:** elastogenesis, M2 pathway, neocollagenesis, skin booster, transparent porcine‐derived atelocollagen

## Abstract

**Background:**

Skin aging is characterized by the loss of collagen and elastic fibers, leading to reduced elasticity and wrinkle formation. Although skin boosters are widely used for skin rejuvenation, direct evidence of elastic fiber regeneration remains limited.

**Aims:**

To evaluate the regenerative effects of transparent porcine‐derived low‐immunogenic Type I atelocollagen in naturally aged skin.

**Methods:**

Transparent porcine‐derived Type I atelocollagen was injected intradermally into the dorsal skin of young (4 months old) and naturally aged (15 months old) mice. Skin tissues were collected 2 and 4 weeks after treatment. Histological analyses (hematoxylin and eosin and Verhoeff staining) were performed to assess skin thickness and elastic fiber regeneration. Macrophage polarization was evaluated by immunohistochemistry, enzyme‐linked immunosorbent assay, and quantitative real‐time PCR. The expression of elastogenesis‐related genes (Eln, Ebp, Fbn1, Fbln5, Lox, Loxl1, Mfap4, and Ltbp4) and skin elasticity in aged mice were also analyzed.

**Results:**

Transparent porcine‐derived Type I atelocollagen significantly increased epidermal and dermal thickness and promoted robust elastic fiber regeneration. Newly formed elastic fibers extended into the upper dermis, restoring a dermal architecture resembling that of young skin. Transient activation of the M2 macrophage pathway was observed during tissue remodeling, with no histological evidence of persistent inflammation at 4 weeks after treatment.

**Conclusions:**

Transparent porcine‐derived Type I atelocollagen enhanced skin thickness, promoted elastic fiber regeneration, and restored youthful dermal architecture while demonstrating favorable biocompatibility through transient M2 macrophage‐mediated tissue remodeling. These findings support the potential of transparent porcine‐derived atelocollagen as an effective skin booster for skin rejuvenation, warranting further investigation of its molecular mechanisms and long‐term clinical efficacy.

## 1 Introduction

Skin aging is a complex biological process characterized by progressive quantitative and qualitative alterations in the extracellular matrix (ECM), including collagen degradation, elastic fiber fragmentation, and impaired matrix remodeling. These changes contribute to reduced skin elasticity, moisture loss, dermal thinning, and wrinkle formation [1]. To address these age‐related alterations, various therapeutic approaches have been developed, including energy‐based devices, injectable biomaterials, and skin boosters that improve dermal quality by promoting collagen synthesis and tissue regeneration [2]. Among these, skin boosters have gained increasing clinical attention as minimally invasive treatments for skin rejuvenation because of their potential to improve skin hydration, elasticity, and overall texture [3]. Atelocollagen, commonly derived from porcine Type I collagen, is produced by enzymatic removal of the non‐helical terminal telopeptide regions, thereby reducing antigenicity while preserving the native triple‐helical collagen structure. As a result, atelocollagen exhibits lower immunogenicity than native collagen while maintaining favorable biocompatibility, biodegradability, cell adhesion properties, and ECM‐mimicking characteristics. These properties have led to its widespread investigation and application in regenerative and aesthetic medicine [4, 5]. Previous studies have suggested that injectable collagen‐based materials may promote dermal remodeling and collagen neosynthesis. However, their effects on elastic fiber regeneration and the associated histological changes following intradermal administration remain poorly understood. A novel transparent porcine‐derived atelocollagen preparation has been introduced for clinical use in tissue augmentation and regeneration in damaged ligaments, tendons, muscles, and fascia. More recently, its application has expanded into aesthetic medicine as a skin booster, reflecting growing interest in collagen‐based injectable therapies for skin rejuvenation. Nevertheless, the biological responses induced by this novel transparent porcine‐derived atelocollagen in aged skin, particularly regarding inflammatory responses, elastic fiber regeneration, and improvements in skin elasticity, have not yet been fully elucidated.

Therefore, the present study investigated the biological effects of intradermal injection of a novel transparent porcine‐derived atelocollagen in a naturally aged mouse model. Histological analyses were performed to evaluate inflammatory responses and elastic fiber regeneration, while skin elasticity was measured to determine whether treatment improved both the structural integrity and functional properties of aged skin.

## 2 Materials and Methods

### 2.1 Experimental Animals and Study Design

Laetigen (PHARVIS KOREA, Seoul, Republic of Korea), a transparent injectable composed of porcine Type I atelocollagen, was used in this study. Male C57BL/6N mice were housed under controlled environmental conditions (20°C–24°C, 45%–55% relative humidity) with free access to food and water. All animal procedures were approved by the Institutional Animal Care and Use Committee (IACUC) of the author's institution. The study was conducted and reported in accordance with the ARRIVE 2.0 guidelines.

**FIGURE 1:**
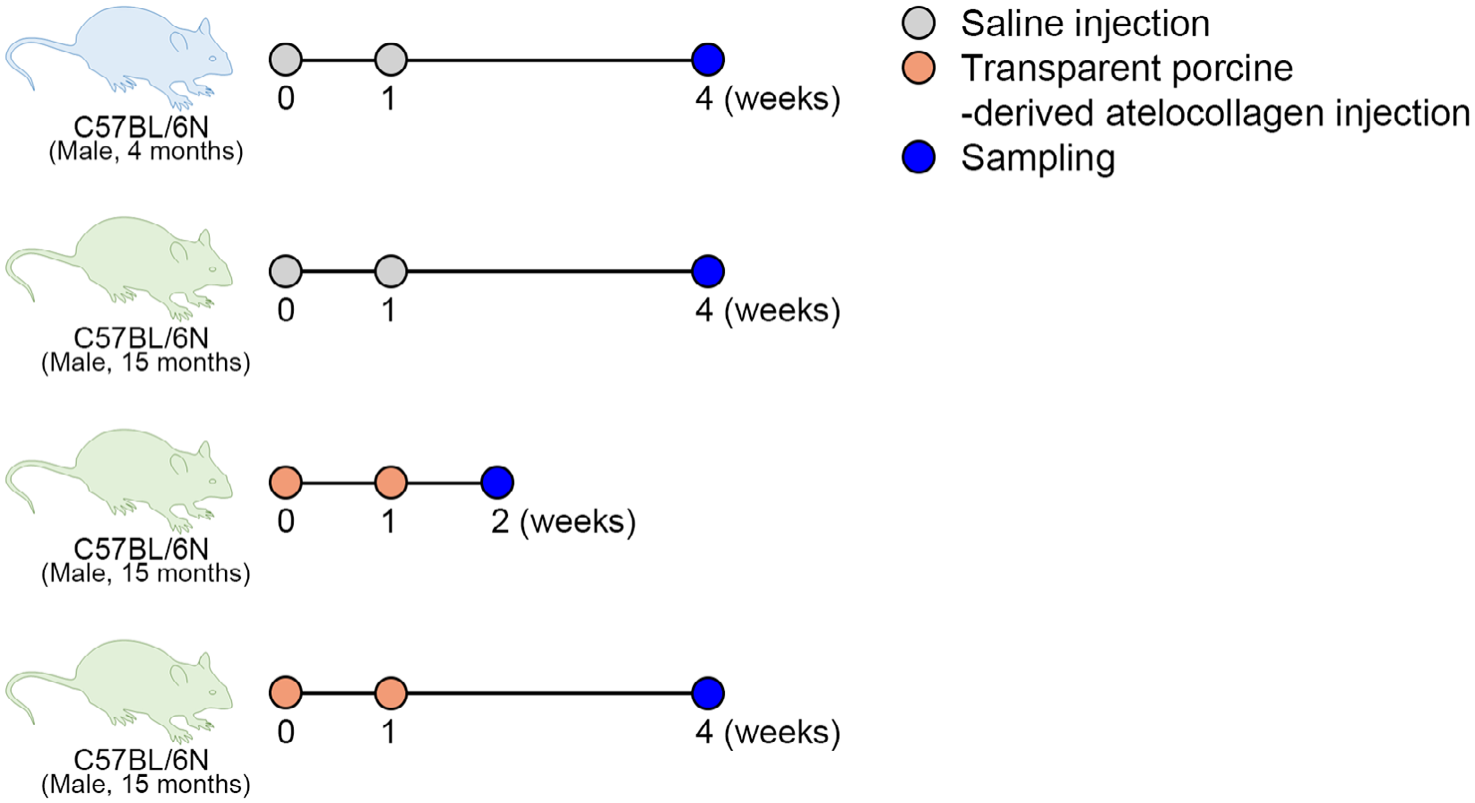
Experimental design of and transparent porcine‐derived atelocollagen injection in aged mice. Group 1 (4‐month‐old mice) and Group 2 (15‐month‐old mice) received saline injections at Weeks 0 and 1, with tissue collection at Week 4. Group 3 (15‐month‐old mice) were treated with porcine Type I atelocollagen at Weeks 0 and 1, and tissues were harvested at Week 2 to assess early effects. Group 4 received the same injections, with tissue collection at Week 4 to evaluate longer‐term effects. Injection and sampling time points are indicated by circles (saline: gray; collagen: orange; sampling: blue).

Young (4 months old) and aged (15 months old) mice were used. Animals were allocated to four experimental groups (Figure 1), with five mice per group. Aged mice were allocated to the three aged groups using a predefined alternating sequence. Animals in the aged groups were allocated using a predefined alternating sequence rather than computer‐generated randomization.

Group 1: Young mice were injected with saline at 0 and 1 week, and tissues were collected at 4 weeks.

Group 2: Aged mice were injected with saline at 0 and 1 week, and tissues were collected at 4 weeks.

Group 3: Aged mice were injected with porcine Type 1 atelocollagen at 0 and 1 week, and tissues were collected at 2 weeks.

Group 4: Aged mice were injected with porcine Type 1 atelocollagen at 0 and 1 week, and tissues were collected at 4 weeks.

### 2.2 Dose Scaling and Intradermal Injection Procedure

The clinical dosing regimen consisted of three 2‐mL injections administered at 1–4‐week intervals (total dose, 6 mL) over a treatment area of approximately 17.7 cm^2^. Assuming linear scaling according to the treatment area to the 4 cm^2^ dorsal treatment area in mice, the equivalent cumulative dose was estimated to be 1.36 mL (6 × 4/17.7). Accordingly, each mouse received a total of 1.4 mL, administered as two 0.7‐mL injections at a 1‐week interval.

Before each injection, mice were anesthetized, and the dorsal hair was completely removed using a razor followed by depilatory cream. At each treatment, 0.7 mL of atelocollagen or saline was divided into multiple small aliquots and injected across multiple sites within the predetermined 4 cm^2^ dorsal treatment area to minimize excessive local swelling. The needle was inserted at a shallow angle with the bevel facing upward to deliver the material into the dermal layer. All injections were manually administered at a consistent slow injection rate by the same investigator. Mice in the saline control groups underwent the identical injection procedure, including the injection volume, dosing schedule, treatment area, distribution of injection sites, and injection technique.

### 2.3 Sample Preparation

#### 2.3.1 Tissue Section

After complete hair removal, full‐thickness dorsal skin samples were harvested. Tissues were frozen in liquid nitrogen for RNA isolation, and skin was fixed in 4% paraformaldehyde (Sigma Aldrich, St. Louis, MO, USA) for paraffin embedding. The skin tissues were fixed in cold 4% paraformaldehyde (Sigma‐Aldrich) for 72 h, placed in cassettes, and washed with distilled water. The tissues were processed in a tissue processor (Leica, Wetzlar, Germany) and sequentially immersed in 95% and 99% ethanol (Duksan, Ansan, Republic of Korea), followed by xylene (Duksan), and finally infiltrated with paraffin (Sakura Finetek Inc., Torrance, CA, USA). Samples were prepared using an embedding machine (Sakura Finetek Inc.). The blocks were cut into 7‐μm‐thick sections using a microtome (Leica), placed on coated slides (MUTO PURE CHEMICALS CO. Ltd., Tokyo, Japan), and incubated at 60°C overnight for attachment.

#### 2.3.2 Protein Isolation

Total proteins were extracted from skin tissue samples using radioimmunoprecipitation assay (RIPA) lysis buffer supplemented with protease and phosphatase inhibitor cocktails. Briefly, skin tissues were washed with ice‐cold phosphate‐buffered saline (PBS) to remove residual blood and debris, minced into small pieces, and homogenized in an appropriate volume of ice‐cold RIPA buffer. The homogenates were incubated on ice for 30 min with intermittent vortexing to ensure complete cell lysis. After centrifugation at 12000–14000 × g for 15–20 min at 4°C, the supernatants containing soluble total proteins were carefully collected and transferred to fresh microcentrifuge tubes. Protein concentrations were determined using a bicinchoninic acid (BCA) protein assay according to the manufacturer's instructions, with bovine serum albumin (BSA) used as the standard. The extracted protein samples were aliquoted and stored at −80°C until further analysis.

#### 2.3.3 RNA Isolation and cDNA Synthesis

Total RNA was isolated using RNAiso reagent (TAKARA, Tokyo, Japan), following the manufacturer's protocol. RNA quantification and quality assessment were performed using a NanoDrop spectrophotometer (Thermo Fisher Scientific, Waltham, MA, USA). cDNA was synthesized using 1 μg of RNA according to the protocol of the PrimeScript 1st strand cDNA Synthesis Kit (TAKARA). Staining.

#### 2.3.4 Immunohistochemistry Staining

To evaluate macrophage infiltration and polarization, immunohistochemical staining was performed using anti‐F4/80 (Santa Cruz Biotechnology, Dallas, TX, USA) and anti‐CD206 (Novus Biologicals, Centennial, CO, USA) primary antibodies. Paraffin‐embedded tissue sections were deparaffinized in xylene, rehydrated through a graded ethanol series, and treated with hydrogen peroxide to block endogenous peroxidase activity. The sections were then incubated overnight at 4°C with the primary antibodies. After washing with PBS, the sections were incubated with horseradish peroxidase (HRP)‐conjugated secondary antibody. Immunoreactivity was visualized using 3,3′‐diaminobenzidine (DAB), followed by counterstaining with hematoxylin.

#### 2.3.5 Verhoeff Staining

Elastic fiber regeneration was evaluated using Verhoeff's elastic fiber staining. Tissue sections were stained according to the manufacturer's instructions using a Verhoeff Elastic Stain Kit (ScyTek Laboratories, Logan, UT, USA).

#### 2.3.6 Hematoxylin and Eosin (H&E) Staining

Histological evaluation was performed on H&E‐stained sections. Tissue specimens were fixed, embedded in paraffin, sectioned, and stained with H&E according to standard histological procedures.

### 2.4 Quantitative Image Analysis

All images were acquired using identical microscope magnification and image acquisition settings. DAB‐positive staining was separated from the hematoxylin counterstain using the Color Deconvolution 2 plugin in ImageJ software (National Institutes of Health, Bethesda, MD, USA), followed by a fixed intensity threshold. For Verhoeff‐stained sections, elastic fiber‐positive signals (dark blue to black) were segmented using the same fixed‐threshold intensity method. An identical threshold value was applied to all samples within the same staining batch. Processing artifacts were manually excluded from the analysis. For each animal, three nonoverlapping high‐power fields (HPFs) were analyzed, and the mean value was calculated to represent a single animal‐level measurement. Quantitative values were normalized to the Young/Saline group and expressed as fold changes.

Epidermal thickness was measured as the distance from the basal layer to the outer surface of the epidermis, whereas dermal thickness was measured from the dermal–epidermal junction to the interface between the dermis and the subcutaneous tissue. Measurements obtained from three nonoverlapping HPFs were averaged to generate a single value for each animal.

### 2.5 Enzyme‐Linked Immunosorbent Assay (ELISA)

An indirect ELISA was performed using equal amounts of total protein coated onto 96‐well microplates and incubated overnight at 4°C. After blocking with 5% skim milk in PBS, the plates were incubated with primary antibodies against inducible nitric oxide synthase (iNOS; Cell Signaling Technology, Danvers, MA, USA) or arginase‐1 (Arg‐1; Santa Cruz Biotechnology, Dallas, TX, USA). After washing with PBS containing 0.05% Tween‐20 (PBST), the plates were incubated with HRP‐conjugated secondary antibodies. Immunoreactivity was detected using 3,3′,5,5′‐tetramethylbenzidine (TMB) substrate, and the reaction was terminated by adding stop solution. Absorbance was measured at 450 nm using a microplate reader.

### 2.6 Quantitative Reverse Transcription Polymerase Chain Reaction (qRT‐PCR)

The synthesized cDNA was diluted 1:2, and qRT‐PCR was performed in a final reaction volume of 10 μL containing 1 μL of diluted cDNA template, 0.4 μL each of forward and reverse primers, 5 μL of ROX Plus SYBR Green Premix (Takara Bio Inc., Shiga, Japan), and 3.2 μL of nuclease‐free water. Amplification and melting curve analyses were performed using a QuantStudio 3 Real‐Time PCR System (Thermo Fisher Scientific, Waltham, MA, USA). The PCR conditions consisted of an initial denaturation at 95°C for 10 min, followed by 40 cycles of denaturation at 95°C for 15 s and annealing/extension at 60°C for 1 min. A melting curve analysis was subsequently performed to verify amplification specificity. Relative mRNA expression levels were calculated using the comparative threshold cycle (2^−ΔΔCt^) method. The expression of each target gene was normalized to *Actb* and expressed relative to Young/Saline group.

### 2.7 Analysis of Inflammatory Response Markers

To evaluate the inflammatory response following Laetigen injection, the mRNA expression levels of macrophage polarization markers, including the inflammatory cytokines Il1b (pro‐inflammatory) and Il10 (anti‐inflammatory), were quantified by qRT‐PCR. Primer sequences are listed in Table 1.

**TABLE 1:** List of primers for quantitative reverse transcription‐polymerase chain reaction.

Gene		Primers
*Actb*	Forward	5′‐CCG TAA AGA CCT CTA TGC CAA C‐3′
	Reverse	5′‐GCA GTA ATC TCC TTC TGC ATC C‐3′
*Cd80*	Forward	5′‐GAC CGA ATC TAC TGG CAA AAA C‐3′
	Reverse	5′‐TTC TTA TAC TCG GGC CAC ACT T‐3′
*Cd163*	Forward	5′‐CAC GGC ACT CTT GGT TTG TG‐3′
	Reverse	5′‐CCC CGA GGA TTT CAG CAA GT‐3′
*Il‐1β*	Forward	5′‐ACC AAG CAA CGA CAA AAT ACC T‐3′
	Reverse	5′‐CCG TCT TTC ATT ACA CAG GAC A‐3′
*Il‐10*	Forward	5′‐TTG ACA TCT CCC CCG GAT CT‐3′
	Reverse	5′‐AAC ACA AGC GTG GAT TTG GC‐3′
*Eln*	Forward	5′‐TAT TAT CCA GGG GCT GGT ATT G‐3′
	Reverse	5′‐CTT AGG TGG TTT TCC TCC AGG T‐3′
*Ebp*	Forward	5′‐CGC ACG GCA TCT ATA ATG TCA C‐3′
	Reverse	5′‐AGA TGT ATC GGA ATG GCT GTC C‐3′

### 2.8 Analysis of Genes Associated With Elastic Fiber Formation

The mRNA expression levels of genes involved in elastic fiber formation, assembly, and maturation were evaluated by qRT‐PCR. The target genes included Eln (Elastin), Ebp (Glb1; Elastin‐binding protein), Fbn1 (Fibrillin‐1), Fbln5 (Fibulin‐5), Lox (Lysyl oxidase), Loxl1 (Lysyl oxidase‐like 1), Mfap4 (Microfibril‐associated protein 4), and Ltbp4 (Latent transforming growth factor beta‐binding protein 4). Primer sequences are provided in Table 1.

### 2.9 Skin Elasticity Measurement

Skin elasticity was measured using an API‐100 skin analyzer (Aram Huvis Co. Ltd., Seongnam, Republic of Korea), and the results were expressed in arbitrary units (A.U.). To minimize measurement variability, all measurements were performed at the same anatomical location and probe orientation under consistent skin hydration conditions. The operator was blinded to the treatment allocation throughout the study. Skin elasticity was assessed immediately before substance injection (baseline) and again immediately before tissue harvesting. At each time point, five consecutive measurements were obtained and averaged. Changes in skin elasticity were calculated as the difference between the post‐treatment value (before tissue harvesting) and the baseline value (before treatment).

### 2.10 Statistical Analysis

Statistical analyses were performed using SPSS version 26.0 (IBM Corp., Armonk, NY, USA). The individual animal was considered the experimental unit. Owing to the small sample size, nonparametric methods were used throughout. Overall differences among the four experimental groups were assessed using the Kruskal–Wallis test.

Four biologically prespecified pairwise comparisons were performed using two‐sided exact Mann–Whitney *U* tests: (1) Young/Saline versus Aging/Saline; (2) Aging/Saline versus Aging/Transparent porcine‐derived atelocollagen (2 weeks); (3) Aging/Saline versus Aging/Transparent porcine‐derived atelocollagen (4 weeks); and (4) Aging/Transparent porcine‐derived atelocollagen (2 weeks) versus Aging/Transparent porcine‐derived atelocollagen (4 weeks).

To control the family‐wise error rate within each outcome, Bonferroni correction was applied across the four prespecified pairwise comparisons. Bonferroni‐adjusted *p* values were obtained by multiplying the exact *p* values by four, with adjusted values truncated at 1.00. Statistical significance was defined as an adjusted *p* value < 0.05. Kruskal–Wallis omnibus *p* values were reported without adjustment. All statistical tests were two‐sided.

Because the study included a fixed sample size of five animals per group, a sensitivity power analysis was performed using G*Power version 3.1 for a two‐sided Wilcoxon–Mann–Whitney test assuming a normal parent distribution. With five animals per group, a Bonferroni‐adjusted significance level (α = 0.0125), and 80% statistical power, the minimum detectable standardized effect size was estimated to be Cohen's d = 2.74. AI disclosure.

Generative artificial intelligence tools were used to assist with English‐language editing, proofreading of typographical errors, and independent verification of the statistical analyses. The authors did not use AI for study design, data generation, primary data analysis, interpretation of results, or the drawing of scientific conclusions. All AI‐assisted outputs were subsequently reviewed, verified, and edited by the authors, who take full responsibility for the content and integrity of the manuscript.

## 3 Results

**FIGURE 2:**
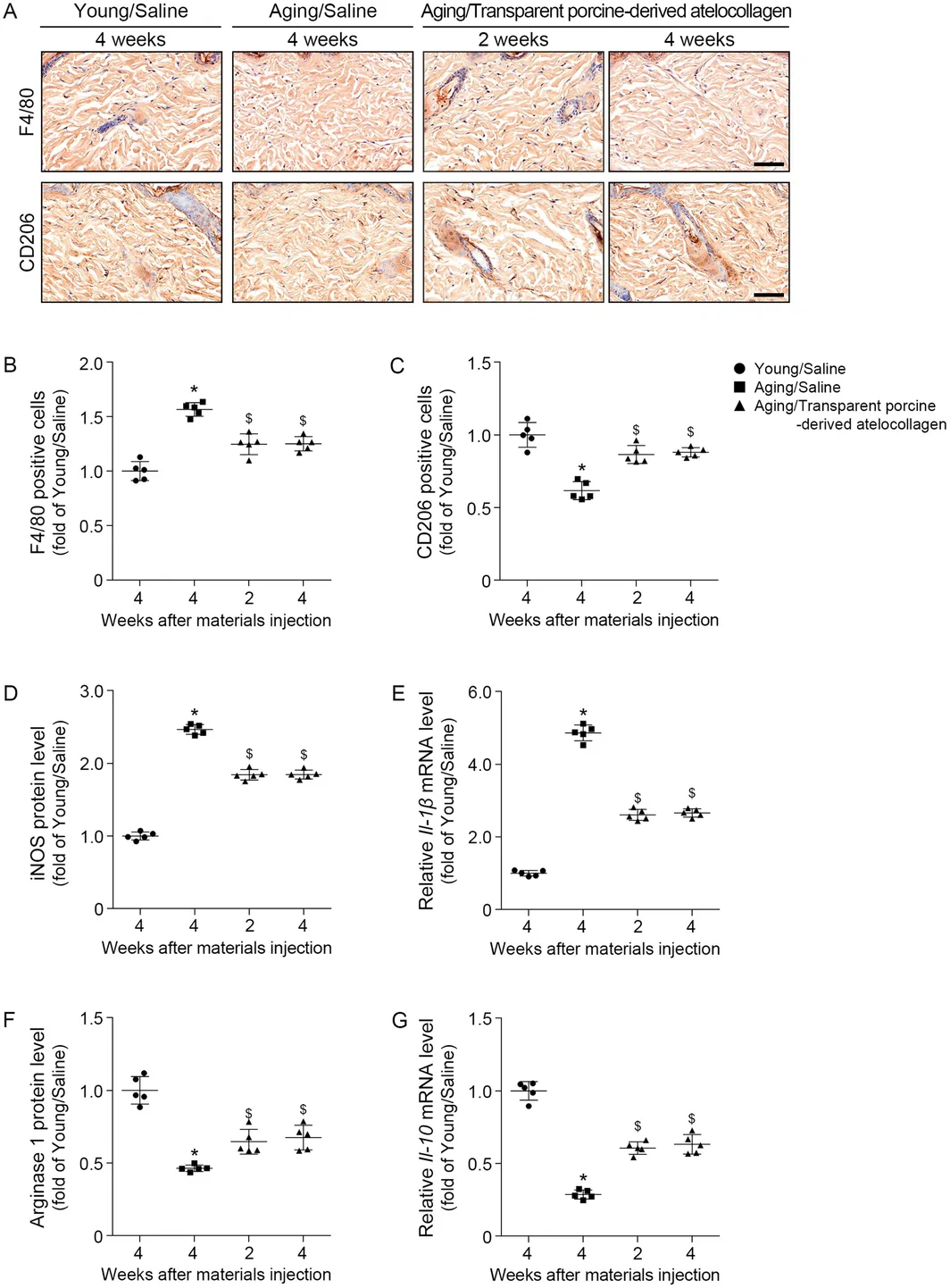
Effect of transparent porcine‐derived atelocollagen on macrophage polarization and inflammation‐related marker expression in aged mouse skin. (A) Representative immunohistochemical images of F4/80‐positive (upper panels) and CD206‐positive (lower panels) macrophages in the dermis of young (4‐month‐old) and aged (15‐month‐old) male C57BL/6N mice at 2 and 4 weeks after intradermal injection of saline or transparent porcine‐derived Type 1 atelocollagen. Scale bar = 50 μm. (B) Quantification of F4/80‐positive cells (pan‐macrophage marker) and (C) CD206‐positive cells (M2 macrophage marker), expressed as fold of the Young/Saline group. (D) iNOS protein level and (E) relative *Il‐1β* mRNA level as M1‐associated markers. (F) Arginase 1 protein level and (G) relative *Il‐10* mRNA level as M2‐associated markers. mRNA expression levels were normalized to *Actb* and presented relative to the Young/Saline group. Each bar represents mean ± SD (*n* = 5 per group). *p < 0.05 vs. Young/Saline group; $ *p* < 0.05 vs. Aging/Saline (Mann–Whitney *U* test).

To investigate the immunomodulatory effects of a novel transparent porcine‐derived atelocollagen, macrophage polarization was evaluated by immunohistochemistry. F4/80‐positive macrophages were markedly increased in aged skin compared with young skin but were significantly reduced following a novel transparent porcine‐derived atelocollagen treatment. In contrast, CD206‐positive M2 macrophages, which were decreased in aged skin, were significantly restored after a novel transparent porcine‐derived atelocollagen administration (Figure 2A–C). Consistent with these findings, the expression of the M1‐associated markers iNOS and Il‐1 was significantly decreased, whereas the expression of the M2‐associated markers Arg1 and Il‐10 was significantly increased following a novel transparent porcine‐derived atelocollagen treatment (Figure 2D–G). These findings indicate that a novel transparent porcine‐derived atelocollagen shifted macrophage polarization from the pro‐inflammatory M1 phenotype toward the anti‐inflammatory M2 phenotype in aged skin.

**FIGURE 3:**
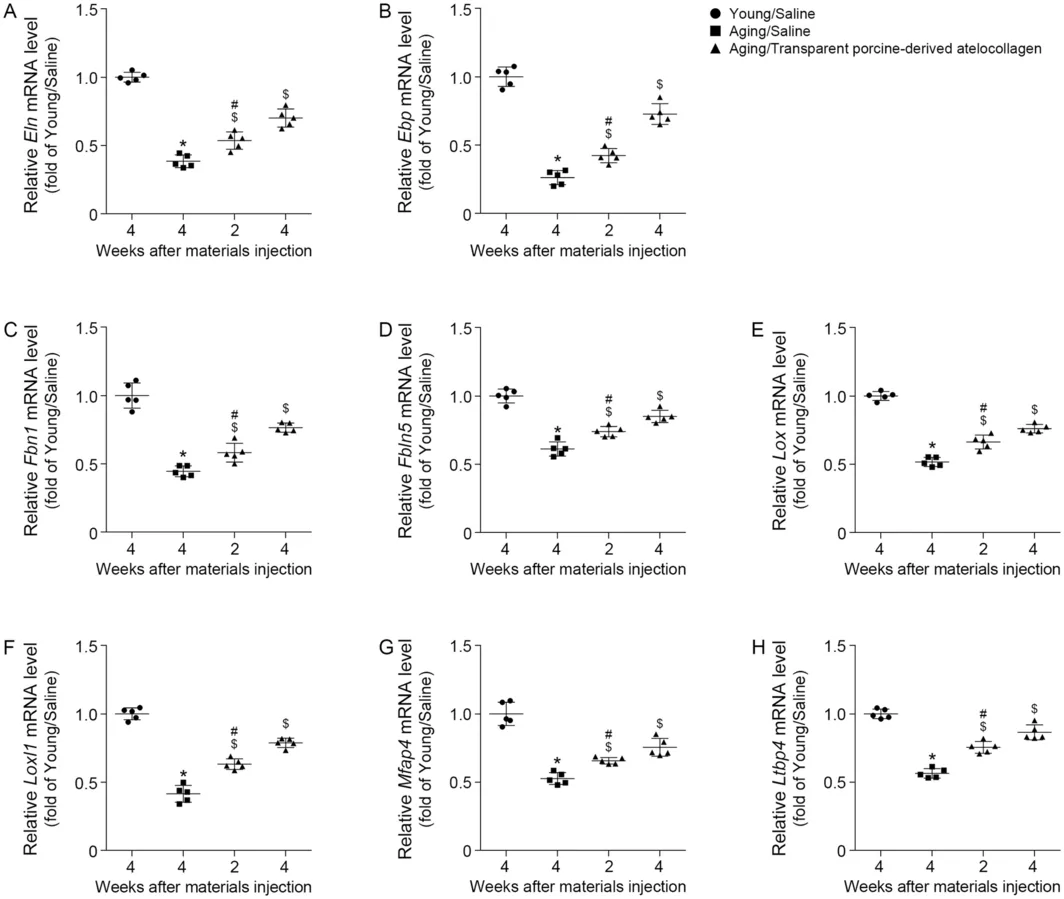
Effect of transparent porcine‐derived atelocollagen on mRNA expression of elastic fiber‐related genes in aged mouse skin. Quantitative RT‐PCR analysis of mRNA levels for (A) *Eln*, (B) *Ebp* (Elastin binding protein; *Glb1*), (C) *Fbn1* (Fibrillin‐1), (D) *Fbln5* (Fibulin‐5), (E) *Lox* (Lysyl oxidase), (F) *Loxl1* (Lysyl oxidase‐like 1), (G) *Mfap4* (Microfibril‐associated protein 4), and (H) *Ltbp4* (Latent TGF‐β binding protein 4) in skin of young (4‐month‐old) and aged (15‐month‐old) male C57BL/6N mice at 2 and 4 weeks after injection of saline or porcine Type I atelocollagen. Gene expression levels were normalized to *Actb* and presented relative to the Young/Saline group. Each bar represents mean ± SD (*n* = 5 per group). *p < 0.05 vs. Young/Saline group; $ *p* < 0.05 vs. Aging/Saline; # *p* < 0.05 Aging/porcine Type I atelocollagen 2 weeks vs. Aging/porcine Type I atelocollagen 4 weeks (Mann–Whitney *U* test).

Compared with the aged saline‐treated control group, the novel transparent porcine‐derived atelocollagen‐treated group showed significantly increased expression of genes involved in elastic fiber formation, including those encoding elastin, elastin‐binding protein, and elastic fiber‐associated molecules (Figure 3).

**FIGURE 4:**
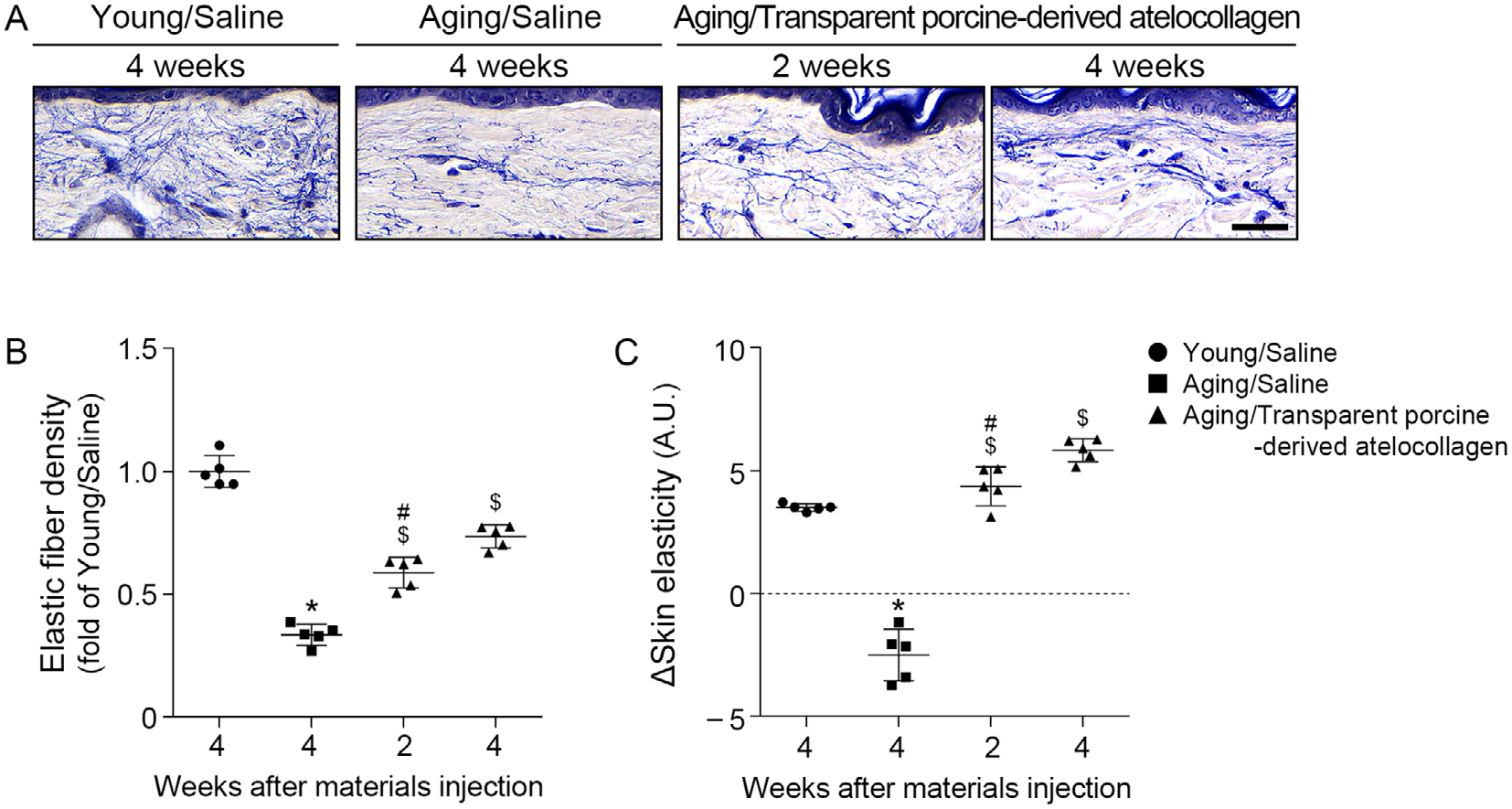
Effect of transparent porcine‐derived atelocollagen on elastic fiber density and skin elasticity in aged mouse skin. (A) Representative images of elastic fibers stained with Verhoeff in skin of young (4‐month‐old) and aged (15‐month‐old) male C57BL/6N mice at 2 and 4 weeks after injection of saline or porcine Type I atelocollagen. Elastic fibers appear as dark blue to black strands within the dermis. Scale bar = 50 μm. (B) Quantification of elastic fiber density relative to the Young/Saline group. (C) In vivo measurement of change in skin elasticity expressed in arbitrary units (A.U.). Each bar represents mean ± SD (*n* = 5 per group). *p < 0.05 vs. Young/Saline group; $ *p* < 0.05 vs. Aging/Saline; # *p* < 0.05 Aging/porcine Type I atelocollagen 2 weeks vs. Aging/porcine Type I atelocollagen 4 weeks (Mann–Whitney *U* test).

Histological analysis demonstrated progressive regeneration of dermal elastic fibers following intradermal administration of a novel transparent porcine‐derived atelocollagen. At 2 weeks after treatment, newly synthesized elastic fibers were detectable within the dermis. By 4 weeks, the density of these fibers had markedly increased, and they exhibited a well‐organized distribution extending toward the dermal–epidermal junction. Quantitative analysis confirmed that elastic fiber density was significantly greater in the novel transparent porcine‐derived atelocollagen‐treated group than in the aged saline‐treated control group (Figure 4A,B).

To determine whether these histological changes were accompanied by functional improvement, skin elasticity was assessed at the indicated time points. The novel transparent porcine‐derived atelocollagen‐treated group exhibited significantly greater skin elasticity than the aged saline‐treated control group throughout the experimental period. Furthermore, skin elasticity progressively increased between Weeks 2 and 4 after treatment (Figure 4C).

**FIGURE 5:**
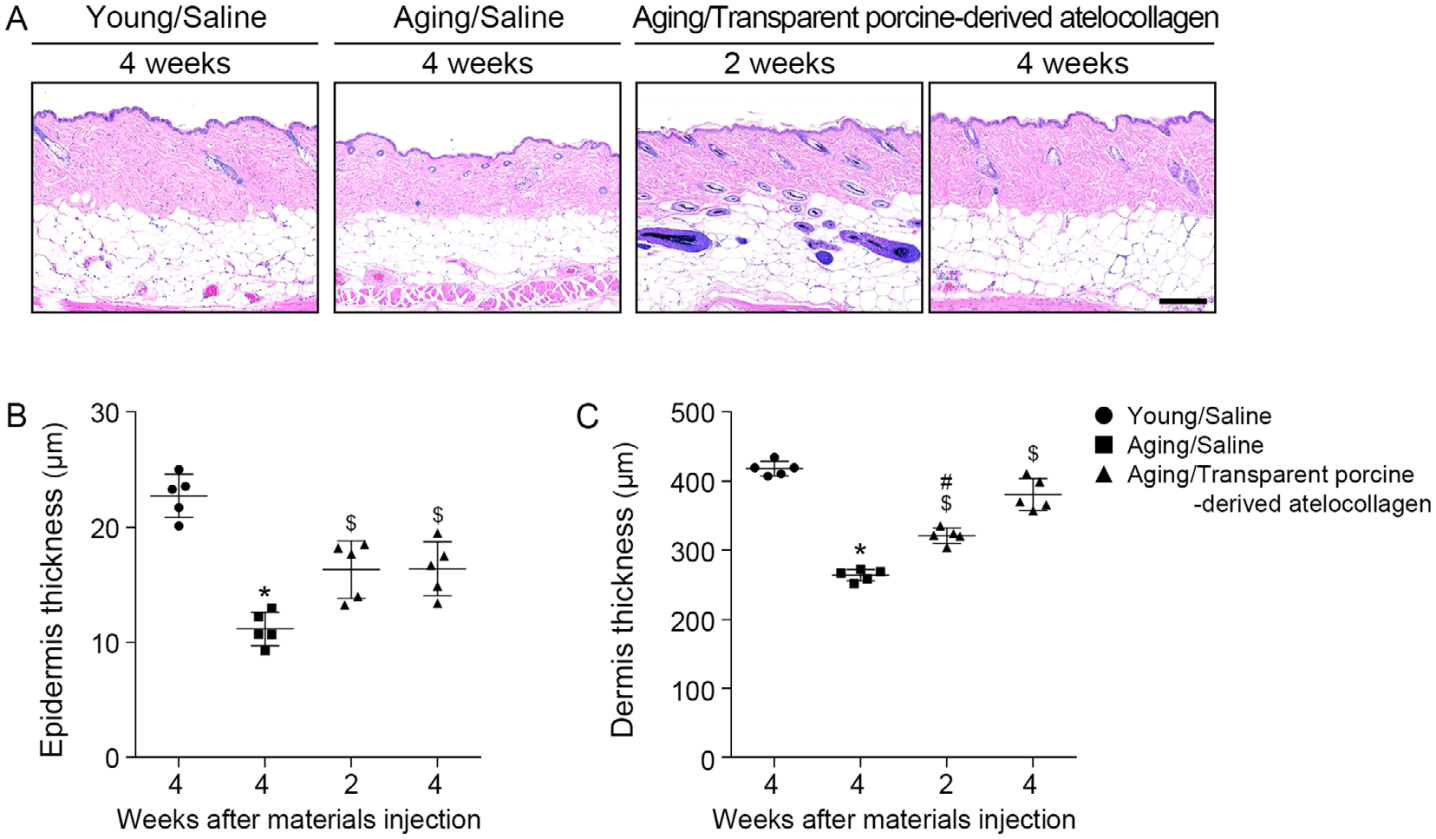
Effect of transparent porcine‐derived atelocollagen on epidermal and dermal thickness in aged mouse skin. (A) Representative images of hematoxylin and eosin stain in skin of young (4‐month‐old) and aged (15‐month‐old) male C57BL/6N mice at 2 and 4 weeks after injection of saline or porcine Type I atelocollagen. Scale bar = 100 μm. (B, C) Quantification of (B) epidermal thickness and (C) dermal thickness. Each bar represents mean ± SD (*n* = 5 per group). *p < 0.05 vs. Young/Saline group; $ *p* < 0.05 vs. Aging/Saline; # *p* < 0.05 Aging/porcine Type I atelocollagen 2 weeks vs. Aging/porcine Type I atelocollagen 4 weeks (Mann–Whitney *U* test).

Histological evaluation of H&E‐stained skin sections demonstrated that intradermal administration of a novel transparent porcine‐derived atelocollagen markedly improved the structural characteristics of aged skin. Compared with the aged saline‐treated control group, both epidermal and dermal thicknesses were significantly increased at 2 and 4 weeks after treatment, indicating progressive restoration of skin architecture (Figure 5).

## 4 Discussion

To our knowledge, this study is among the first to investigate the in vivo effects of intradermally injected transparent porcine‐derived Type I atelocollagen on elastic fiber regeneration, macrophage polarization, and skin elasticity in naturally aged skin. Treatment induced progressive regeneration of dermal elastic fibers, including vertically oriented fibers extending toward the dermal–epidermal junction, together with increased epidermal and dermal thickness. These structural changes were accompanied by significant improvements in skin elasticity, suggesting coordinated remodeling of the aged dermal extracellular matrix.

Skin aging is associated with chronic low‐grade inflammation, reduced fibroblast regenerative capacity, and impaired elastogenesis. Previous studies have suggested that inflammatory responses, particularly macrophage polarization, influence extracellular matrix remodeling and elastic fiber formation. Horiba et al. reported that a reduction in M2 macrophages increased the M1/M2 ratio, enhanced matrix metalloproteinase activity, and was associated with abnormal collagen organization [6]. Similarly, Gu et al. demonstrated that reduced AKT3 expression in M2 macrophages was associated with impaired collagen and elastic fiber production in chronic wounds [7]. Oh et al. also reported that poly‐L‐lactic acid (PLLA) fillers promoted M2 macrophage activation, which was accompanied by elastic fiber regeneration in aged skin [8]. In the present study, intradermal administration of transparent porcine‐derived Type I atelocollagen reduced total macrophage infiltration while promoting polarization toward the M2 phenotype, as demonstrated by increased CD206 expression together with upregulation of Arg1 and Il‐10 and downregulation of iNOS and Il‐1. These findings suggest that modulation of the inflammatory microenvironment may facilitate extracellular matrix remodeling and create conditions favorable for elastic fiber regeneration.

Histologically, aging skin is characterized not only by a reduction in elastic fibers but also by the loss of vertically oriented elastic fibers extending toward the basement membrane [9]. Because elastic fiber regeneration requires coordinated assembly of elastin with multiple microfibrillar proteins, it represents a more complex biological process than elastin synthesis alone [10, 11]. Elastogenesis involves sequential processes, including tropoelastin secretion, coacervation, cross‐linking, and incorporation into the microfibrillar scaffold, all of which are required to establish a functional elastic fiber network [12, 13, 14]. Therefore, evaluation of elastic fiber architecture, rather than elastin expression alone, may provide a more comprehensive assessment of functional tissue remodeling following regenerative therapies because proper fiber organization is essential for the biomechanical function of elastic tissue.

In addition to increased elastic fiber formation, the present study demonstrated increased epidermal and dermal thickness after treatment. In parallel with these histological findings, skin elasticity progressively improved throughout the observation period. Because dermal elastic fibers are major determinants of tissue recoil and mechanical resilience, the functional improvement observed in this study is consistent with the histological evidence of elastic fiber regeneration, although direct causality cannot be established from the present data. These findings are consistent with enhanced extracellular matrix remodeling, although the specific cellular and molecular mechanisms remain to be fully elucidated. Although the underlying cellular mechanisms were not directly investigated. Together, these histological changes suggest that transparent porcine‐derived Type I atelocollagen treatment may promote structural remodeling of aged skin.

Injectable skin boosters have been increasingly used for the treatment of skin aging and wrinkles [15]. The present study extends previous work by demonstrating not only increased expression of elastogenesis‐related genes but also histological evidence of progressive elastic fiber regeneration accompanied by improvements in skin elasticity. These findings support the biological activity of transparent porcine Type I atelocollagen in aged skin. However, whether these histological changes translate into superior clinical outcomes remains to be established through comparative and prospective clinical studies.

Several limitations should be considered when interpreting the present findings. First, although an aged saline‐treated control group was included, no active comparator such as hyaluronic acid‐ or PLLA‐based skin boosters was evaluated. Consequently, it remains uncertain whether the observed biological responses are unique to Type I atelocollagen, represent nonspecific tissue responses to injected biomaterials, or are partially attributable to mechanical stimulation associated with the injection procedure. Future studies incorporating both placebo and active comparator groups are needed to clarify the relative biological effects of atelocollagen.

Second, the observation period was relatively short because the estimated half‐life of a novel transparent porcine‐derived atelocollagen is approximately 6 weeks. Therefore, evaluation beyond the degradation period would be particularly informative for determining whether tissue remodeling persists after material resorption. Although progressive elastic fiber formation and M2 macrophage activation were observed during the study period, the long‐term persistence and functional significance of these changes remain unknown. Longer follow‐up studies are warranted to evaluate the durability of tissue remodeling and to further investigate the underlying biological mechanisms.

Third, although the present molecular findings support elastogenic activity, they do not directly demonstrate de novo elastic fiber network assembly because Verhoeff–Van Gieson staining alone cannot distinguish newly synthesized fibers from remodeling of pre‐existing elastic fibers. More definitive validation would require immunohistochemical evaluation of elastin and fibrillin‐1, together with quantitative analyses of fiber morphology and network organization, including fiber length, orientation, branching, and architectural complexity. These investigations were beyond the scope of the present study but represent important priorities for future research to confirm the structural maturation and functional integration of newly formed elastic fibers.

Fourth, the sensitivity analysis indicated that the present study had 80% statistical power to detect only very large between‐group differences (standardized effect size, d = 2.74). Therefore, smaller but potentially biologically or clinically relevant effects may not have been detected. Studies with larger sample sizes are needed to validate these findings and more precisely estimate treatment effects. Accordingly, the possibility of Type II error for more modest treatment effects cannot be excluded.

Finally, future studies may benefit from incorporating AI‐assisted image analysis to enable objective, reproducible, and quantitative assessment of dermal architecture and elastic fiber organization. Integration of AI‐based image analysis with conventional histological and molecular approaches may further improve longitudinal evaluation of tissue remodeling following regenerative therapies [16, 17].

In conclusion, intradermal injection of transparent porcine Type I atelocollagen promoted elastic fiber regeneration, increased epidermal and dermal thickness, and shifted macrophage polarization toward an anti‐inflammatory M2 phenotype in naturally aged mice. These structural and immunological changes were accompanied by improved skin elasticity, suggesting that atelocollagen creates a tissue microenvironment favorable for extracellular matrix remodeling. Although the precise mechanisms and long‐term clinical relevance remain to be established, the present findings provide experimental evidence supporting the regenerative potential of collagen‐based skin boosters for skin rejuvenation.

## Author Contributions

S.M.O., M.S.K., G.K., and M.O. performed the research. S.M.O., M.S.K., G.K., and M.O. designed the research study. S.M.O. and M.S.K. contributed essential reagents and tools. S.M.O. and M.O. analyzed the data. S.M.O., M.S.K., G.K., and M.O. wrote and revised the manuscript. All authors read and approved the final version of the manuscript.

## Funding

This work was supported by Pharvis KOREA Inc.

## Ethics Statement

All animal procedures were approved by the Institutional Animal Care and Use Committee (IACUC approval no.: LCDI‐2025‐0001) and were performed in accordance with national animal‐welfare regulations. The manuscript is reported in accordance with the ARRIVE 2.0 reporting guidelines (completed checklist provided as Supporting Information). Human data were not involved in this study.

## Conflicts of Interest

Seung Min Oh, Min Seong Kim, Geebum Kim, and Myungjune Oh are advisory board members of PHARVIS KOREA Co. Ltd. The authors declare no other financial or nonfinancial competing interests. This relationship was disclosed to the Editor upon submission and is repeated here in full for transparency.

## Data Availability

All quantitative data supporting the findings of this study—including per‐animal Verhoeff densitometry, H&E‐derived epidermal/dermal thickness, IHC cell counts, ELISA absorbance, qRT‐PCR ΔΔCt values, and API‐100 elasticity readings—are provided in the Supporting Information file accompanying this submission and are additionally available from the corresponding author on reasonable request.
